# A Novel Role for the GTPase-Activating Protein Bud2 in the Spindle Position Checkpoint

**DOI:** 10.1371/journal.pone.0036127

**Published:** 2012-04-25

**Authors:** Scott A. Nelson, Anthony M. Sanson, Hay-Oak Park, John A. Cooper

**Affiliations:** 1 Department of Cell Biology and Physiology, Washington University, Saint Louis, Missouri, United States of America; 2 Department of Molecular Genetics, The Ohio State University, Columbus, Ohio, United States of America; Duke University Medical Center, United States of America

## Abstract

The spindle position checkpoint (SPC) ensures correct mitotic spindle position before allowing mitotic exit in the budding yeast *Saccharomyces cerevisiae*. In a candidate screen for checkpoint genes, we identified *bud2Δ* as deficient for the SPC. Bud2 is a GTPase activating protein (GAP), and the only known substrate of Bud2 was Rsr1/Bud1, a Ras-like GTPase and a central component of the bud-site-selection pathway. Mutants lacking Rsr1/Bud1 had no checkpoint defect, as did strains lacking and overexpressing Bud5, a guanine-nucleotide exchange factor (GEF) for Rsr1/Bud1. Thus, the checkpoint function of Bud2 is distinct from its role in bud site selection. The catalytic activity of the Bud2 GAP domain was required for the checkpoint, based on the failure of the known catalytic point mutant Bud2^R682A^ to function in the checkpoint. Based on assays of heterozygous diploids, *bud2^R682A^*, was dominant for loss of checkpoint but recessive for bud-site-selection failure, further indicating a separation of function. Tem1 is a Ras-like protein and is the critical regulator of mitotic exit, sitting atop the mitotic exit network (MEN). Tem1 is a likely target for Bud2, supported by genetic analyses that exclude other Ras-like proteins.

## Introduction

Eukaryotic cells partition their chromosomes between mother and daughter cells on cell division. In budding yeast, the plane of cell division is determined at the start of the cell cycle by selection of a site of polarized growth - the bud site. As the bud grows, the bud site becomes the position of the mother-bud neck. At anaphase, the mitotic spindle must be perpendicular to and intersect to the plane of cell division, and this requires that one spindle pole body (SPB) move into the bud while the other remains in the mother. To position the mitotic spindle, cytoplasmic (astral) microtubules interact with the bud cortex and pull the spindle into the mother/bud neck, via the independent actions of the dynein and Kar9 pathways [1]. When either the dynein or the Kar9 pathway is defective, the mitotic spindle fails to enter the neck in a timely manner in a fraction of cells. In these cells, the cell cycle arrests, in late anaphase, with the chromosomes near the poles and the spindle microtubules intact. This arrest appears to result from a checkpoint mechanism termed the spindle position checkpoint (SPC) [2]–[5].

To investigate the molecular mechanisms of the spindle position checkpoint, with a focus on the molecular basis by which the cell senses spindle position, we screened candidate null mutants lacking proteins with structural or functional connections to the mother-bud neck. To activate the checkpoint, we performed the screen in a strain lacking dynein function (a dynactin/*arp1Δ* mutant). In such a strain, a fraction of the cells in the population fail to move the spindle into the neck, and the cell cycle halts. When the checkpoint mechanism is defective, these cells proceed to exit from mitosis with the spindle in the mother. In our screen, a *bud2Δ* null mutant gave a relatively strong phenotype, so we have investigated the role of Bud2 in the spindle position checkpoint.

*BUD* genes are so-named because null mutants are defective in bud site selection [6]. At the beginning of the cell cycle, **a** and α cells (such as normal haploids) select a bud site adjacent to the previous budding site [7]. Subsequent sites are chosen by the same rule, in an axial pattern. Diploid **a**/α cells have a bipolar budding pattern, in which the newly born daughter places her first bud site at the distal pole, away from its mother [7], [8]. Subsequent buds form at one pole or the other, chosen at random. Axial and bipolar pathways both require Rsr1/Bud1, a Ras-related protein, as well as Bud2, a GAP for Rsr1/Bud1, and Bud5, a GEF for Rsr1/Bud1 [6], [9]–[12]. Null mutants lacking any of the three proteins show a random pattern of bud site selection, as haploids or diploids, suggesting that Rsr1/Bud1 cycles between GTP and GDP-bound states. Bud2 and Bud5 are recruited to the presumptive bud site by spatial landmarks, and they recruit Rsr1/Bud1 [13]–[15].

Exit from mitosis appears to be controlled by the activity of another Ras-like GTPase, Tem1 [3], [4], [16]. Studies of the mitotic exit network (MEN) identify a putative GAP, composed of Bub2 and Bfa1, and a putative GEF, Lte1. Loss of Bub2 or Bfa1 causes a complete failure of the spindle position checkpoint, as does overexpression of Lte1, which means that 100% of cells with mis-positioned spindles proceed to premature exit from mitosis. Mutations of a pathway that inhibits Lte1 are sufficient to cause failure of the checkpoint, but only in about half of such cells [2]. In these strains, adding an *lte1* null mutation prevents the premature mitotic exit. This pathway also involves a *BUD* gene, *BUD6*.

Here, we show that loss of Bud2 also causes failure of the checkpoint in about half of cells with mis-positioned spindles. The checkpoint defects caused by point mutations affecting the Bud2 GAP domain were similar to the defects caused by null mutations of *bud2*. However, checkpoint failure in the *bud2* mutant was independent of Lte1, providing a contrast to the pathway that involves Bud6 [2]. Remarkably, Rsr1/Bud1, the only known substrate of Bud2, had no role in the SPC, based on several observations.

## Results

### Checkpoint Defects in a bud2Δ Mutant

**Figure 1:**
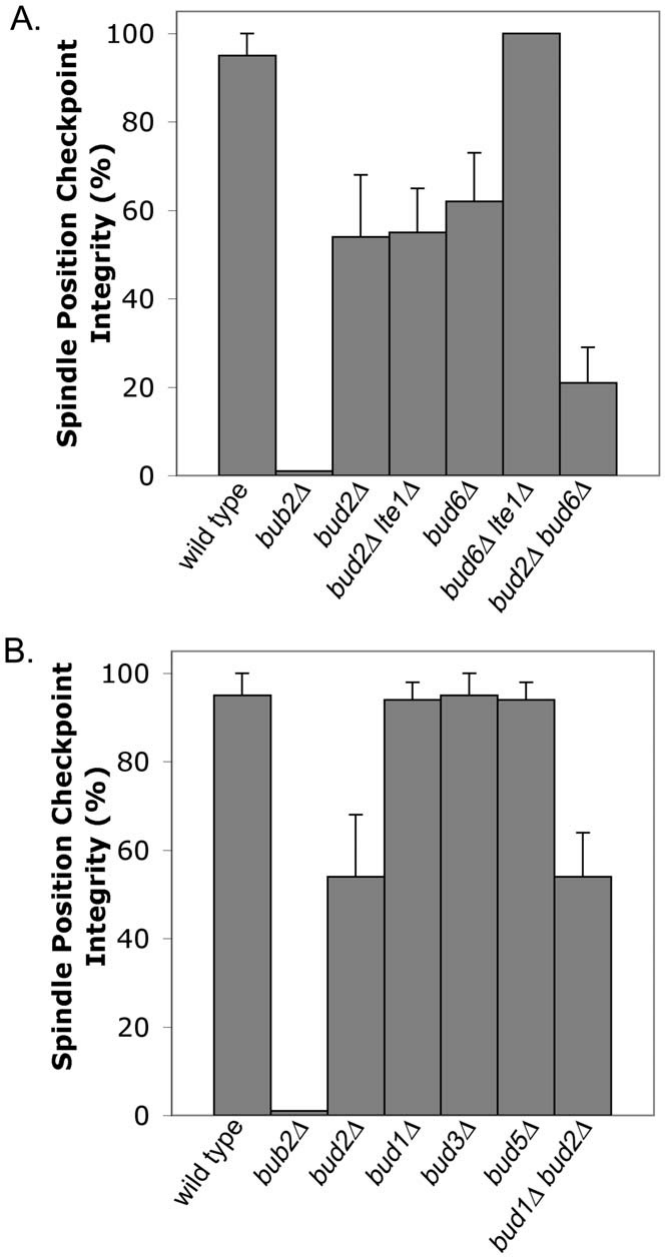
*BUD2* is required for the spindle position checkpoint. *arp1Δ GFP-TUB1* cells with the additional indicated mutations were assayed for checkpoint integrity by video analysis. Cells with long (late-anaphase) spindles in the mother of a budded cell were followed over time. Checkpoint integrity is the percent of cells in which the spindle that remained intact, i.e. did not break down, for a time greater than the mean plus two standard deviations of the time for normal mitotic exit. A. *bud2Δ* mutants have a defect in the spindle position checkpoint, with failure to maintain arrest in about half of cells. *bub2Δ* is a positive control known to have a complete defect. The *bud2Δ* phenotype does not depend on *LTE1*, based on the *bud2Δ lte1Δ* double mutant. *BUD6* is in a pathway upstream of *LTE1*, as described previously [2] and confirmed here. The *bud2 bud6* double mutant has an exacerbated phenotype, confirming that *BUD2* is in a genetic pathway independent of *BUD6* and *LTE1*. The *bud2Δ bud6Δ* double mutant does not have a complete loss of phenotype, as *bub2* does, suggesting a possible third input into the checkpoint control of mitotic exit. B. The bud-site-selection pathway has no role in the spindle position checkpoint. Mutants lacking either Rsr1/Bud1, the only known substrate of Bud2, or Bud5, the GEF for Rsr1/Bud1, have no checkpoint defect. Deleting *RSR1/BUD1* does not suppress the checkpoint defect of a *bud2Δ* mutant. *bud3* and *bud5* null mutants, defective in axial and all budding patterns, respectively, are also normal.

In our screen for candidate checkpoint genes, dynactin/*arp1Δ bud2Δ* mutants had many cells with greater than three nuclei, consistent with but not specific for a defect in the SPC. To assay the integrity of the checkpoint, we performed time-lapse movie analysis of single cells with mitotic spindles in the mother. Using GFP-tubulin as a marker for mitotic spindle breakdown, we calculated the percentage of cells in which the checkpoint was intact (Fig. 1A). About 45% of *bud2Δ* cells with long-late anaphase spindles in the mother displayed a failure of checkpoint-induced arrest, with mitotic spindle breakdown occurring within a time comparable to the time to mitotic exit in an otherwise wild-type (wt) cell. The *bud2*Δ mutants underwent inappropriate spindle breakdown in the mother with a MT in the mother-bud neck, eliminating the possibility that the *bud2*Δ mutant accumulates multinucleate cells as a result of aberrant MT dynamics. An example is shown in Movie S1. A *bud2 lte1* double mutant had a checkpoint defect similar to that of a *bud2* single mutant, indicating that the checkpoint defect was independent of Lte1.

In a previous study, null mutations in a pathway including *BUD6* had a checkpoint defect with magnitude similar to the one observed here for *bud2*, and the *bud6* checkpoint defect did depend completely on *LTE1* [2]. Here, we confirmed those results, and we found that a *bud2*Δ *bud6*Δ double mutant showed an exacerbated checkpoint defect (Fig. 1A), supporting the idea that Bud2's checkpoint function belongs to a molecular pathway distinct from that of Bud6 and Lte1.

### Functional Checkpoint in Other Bud-site Selection Mutants

Bud2 is known to function in the bud-site-selection pathway, acting as a GAP for Rsr1/Bud1 [10]. To determine whether Bud2's action in the SPC involves Rsr1/Bud1 or other bud-site-selection pathway components, we assayed the checkpoint in bud-site selection mutants (Fig. 1B). First, to test whether hyperactive Rsr1/Bud1 resulting from deletion of its GAP is involved in the loss of checkpoint, an *rsr1Δ bud2Δ* double mutant was evaluated and found to be similar to *bud2Δ*. That is, deleting *RSR1* did not suppress the checkpoint defect of a *bud2Δ* mutant. Second, a *rsr1Δ* single mutant had no checkpoint defect. A *bud5Δ* mutant, which lacks the GEF Bud5, had no checkpoint defect. Bud3 and Bud4 are parts of the positional marker for the axial budding pattern in haploid cells; a *bud3Δ* mutant and a *bud4Δ* mutant also had normal checkpoint function. Together, these results suggest that the role of Bud2 in the checkpoint is independent of the bud-site-selection pathway and Rsr1/Bud1.

### The GAP Function of Bud2

**Figure 2:**
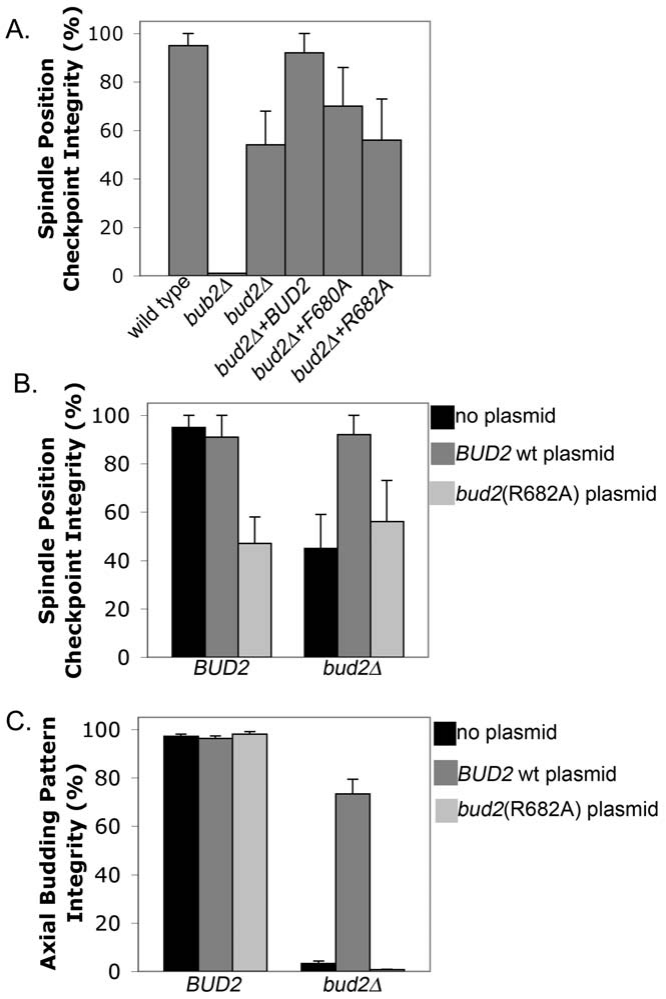
The GAP activity of Bud2. **A.** *arp1Δ GFP-TUB1* cells were assayed as in Figure 1. Wt *BUD2* suppressed the checkpoint defect of a *bud2Δ* mutant, but the GAP-defective point mutant *bud2^R682A^* did not. A temperature-sensitive allele, *bud2^F680A^*, at a semi-permissive temperature (30°), partially suppressed the checkpoint defect. **B.** The checkpoint defect of *bud2^R682A^* was dominant to wt *BUD2*, and *bud2^R682A^* did not enhance the checkpoint defect of a *bud2Δ* mutant. The phenotype of *bud2^F680A^* was not strong, and tests for dominance were inconclusive (data not shown). **C.** Cells with the wt or mutant plasmid were fixed and stained with Calcofluor to observe the budding pattern. Axial budding pattern integrity represents the percentage of cells with multiple bud scars that were all adjacent. The GAP-defective *bud2^R682A^* did not disrupt the bud-site selection pattern of cells with wt *BUD2*, and *bud2^R682A^* does not rescue the bud-site-selection defect of a *bud2Δ* null mutant. In this experiment, cells with two or more bud scars were counted. In an independent experiment, cells with three or more bud scars were counted, and the results were very similar. The only difference was that the value for the *BUD2* wt plasmid was ∼90% instead of ∼70%.

The Bud2 GAP domain contains the ‘FLR motif’, which is highly conserved in Ras-GAPs from other organisms [10]. The arginine residue at position 682 of Bud2 is analogous to Arg at 903 in p120-GAP that is responsible for catalyzing GTP hydrolysis [17], [18]. We constructed two point mutations in the Bud2 GAP domain, *bud2^R682A^* and *bud2^F680A^*, by substituting Arg at 682 to Ala and Phe at 680 to Ala. The *bud2^R682A^* mutant on a low-copy-number CEN plasmid did not rescue the bud-site selection defect of a *bud2* strain (Fig. 2C). The *bud2^F680A^* mutation resulted in a temperature-sensitive bud-site selection defect, and also conferred temperature-dependent synthetic lethality to the *cln1 cln2* mutant (data not shown), suggesting that these *bud2* mutants are defective in the GAP activity.

We tested the checkpoint function of these *bud2* mutant alleles on a low-copy-number CEN plasmid. The *bud2^R682A^* allele did not rescue checkpoint function at all (Fig. 2A), and the temperature-sensitive *bud2^F680A^* allele gave partial rescue at the semi-permissive temperature of 30°. As a control, the wt *BUD2* plasmid gave complete rescue. Thus, the GAP activity of Bud2 is likely to be necessary for its role in the SPC. A *BUD2* strain with the *bud2^R682A^* CEN plasmid had a checkpoint phenotype similar to that of a *bud2* null mutant, indicating that *bud2^R682A^* was dominant for checkpoint function (Fig. 2B). To determine whether *bud2^R682A^* was dominant for the function of bud-site selection, we examined the budding patterns of the same strain (Fig. 2C). Remarkably, *bud2^R682A^* had no effect on bud-site selection in a strain with wt *BUD2*. Thus, *bud2^R682A^* is recessive for bud site selection, but dominant for checkpoint function. The same strains were used for the checkpoint assays and the bud-site-selection assays, arguing against a difference in plasmid copy number or the Bud2 expression level accounting for the difference in dominance. Note that the *bud2^R682A^* plasmid did not exacerbate the checkpoint defect of the *bud2Δ* null mutant, suggesting that the effect of *bud2^R682A^* in a wt cell is due to loss of Bud2 function, not to extraneous effects.

## Discussion

We have discovered that Bud2 is required for the spindle position checkpoint, and this function is independent of bud-site selection. The GAP activity of Bud2 is necessary for checkpoint function and bud-site selection; however, Rsr1/Bud1 and Bud5, which are the small G-protein target of Bud2 and the GEF opposing Bud2, respectively, have no checkpoint function. These results suggest that Bud2 is acting as a GAP for a novel target. Another line of evidence supporting these two independent functions of *BUD2* is the observation that a point mutation in the Bud2 GAP domain is dominant-negative for checkpoint function but recessive for bud-site selection.

### Potential Roles for Bud2 in Checkpoint Pathways

The pathways that control the SPC center on the small G-protein Tem1. The regulator Lte1 had been considered to be a putative GEF for Tem1, but recent reports do not find such activity [19], and instead they argue that Lte1 functions via effects on Bfa1 [19] and Kin4 [20]. We found Lte1 to be downstream of MT-cortex interactions involving a different bud-site selection gene, *BUD6* [2]. Here, we found that the actions of *BUD2* and *BUD6* in the checkpoint are independent *in vivo*. The *bud2* checkpoint phenotype does not depend on *LTE1*, but the checkpoint phenotype of *bud6* does depend on *LTE1*. The *bud2Δ bud6Δ* double null mutant has a more severe checkpoint phenotype than does either single null mutant. Interestingly, the loss of checkpoint in the *bud2Δ bud6Δ* double mutant is not 100%, which can be seen in strains that overexpress *LTE1* or completely lack Bub2, the putative GAP for Tem1. This observation suggests the possible existence of additional, as yet undiscovered, input mechanisms for activating the checkpoint. At this point, nothing is known about what may lie upstream of Bud2 in a checkpoint-related pathway. The nature of this and other potential sensor mechanisms remain to be defined.

Four Ras-like GTPases have been identified in budding yeast: Rsr1/Bud1, Ras1, Ras2, and Tem1. Rsr1/Bud1, a central component of the bud-site-selection pathway, has been eliminated as having a role in the SPC by genetic analyses. Ras1 and Ras2 are believed to have some level of redundancy in their function, which includes cAMP signaling, stress response, and anchoring the MEN activator Lte1 to the bud cortex [21]–[24]. Ras1 and Ras2 are required for the checkpoint, based on results with single null mutants, and, for both, their checkpoint phenotype depends on *LTE1* [2]. Bud2 does not catalyze the hydrolysis of guanine nucleotide bound to Ras2 *in vitro*, but Bud2 does have GAP activity for Rsr1/Bud1 (Figure S1 and [10]). Therefore, we speculate that the substrate of Bud2 for its checkpoint function may be Tem1, which is a potent activator of mitotic exit. Deleting *BUB2/BFA1*, the known GAP for Tem1, yields a strong spindle position checkpoint defect. An alternative hypothesis is that Bud2 inhibits mitotic exit, when the checkpoint is active, through another small GTPase such as a Rab.

The *BUD2* GAP-deficient allele *bud2^R682A^* is dominant for checkpoint function but recessive for bud-site selection. The dominance may result from the mutant form of Bud2 binding to a substrate, such as Tem1, with higher affinity than does wt Bud2, which may result from failure of GTP hydrolysis. The off rate constant may depend on the nucleotide-bound state of the substrate. For Rsr1/Bud1, the substrate for Bud2 in bud site selection, the dependence of the off rate on nucleotide may be different. Alternatively, if the amounts of the two different substrates in cells are different, then the mutant Bud2 may be able to titrate out the one present in a lesser amount but not the one present in a higher amount.

In conclusion, the GAP Bud2 is necessary for the spindle position checkpoint, which is a novel role for Bud2. This function of Bud2 is independent of its role in bud site selection, based on the observations that other *bud* mutants have a normal checkpoint and that a GAP-defective point mutant of Bud2 is dominant for the checkpoint but recessive for bud-site selection.

## Materials and Methods

Reagents were from Fisher Scientific (Pittsburgh, PA) or Sigma-Aldrich (Saint Louis, MO), except as indicated. Yeast were cultured and manipulated by standard techniques [25]. Yeast strains are listed in Table 1.

**Table 1:** List of yeast strains.

Name	YJC #	Relevant genotype
*arp1Δ bub2Δ*	2667	*MAT* ***a*** * arp1Δ::kanR bub2Δ::HIS3 ura3-52 lys2-801 leu2-1 his3-200 trp1-63 ura3-52::URA3-GFP-TUB1*
*arp1Δ bud2Δ*	3560	*MAT* ***a*** * LEU2::LEU2-GFP-TUB1 arp1Δ::KanR bud2Δ::HIS3MX6 ura3-52 lys2-801 leu2-1 his3-Δ200 trp1-63*
*arp1Δ bud3Δ*	3627	*MATα LEU2::LEU2-GFP-TUB1 arp1Δ::KanR bud3Δ::KanR ura3-52 lys2-801 leu2-1 his3-Δ200*
*arp1Δ bud1Δ*	3632	*MAT* ***a*** * LEU2::LEU2-GFP-TUB1 arp1Δ::KanR bud1Δ::KanR ura3-52 lys2-801 leu2-1 his3-Δ200*
*arp1Δ bud2Δ lte1Δ*	3633	*MAT* ***a*** * LEU2::LEU2-GFP-TUB1 arp1Δ::KanR lte1Δ::KanR bud2Δ::HIS3 ura3-52 lys2-801 leu2-1 his3-Δ200*
*arp1Δ bud6Δ lte1Δ*	3635	*MAT* ***a*** * LEU2::LEU2-GFP-TUB1 arp1Δ::KanR lte1Δ::KanR bud6Δ::HIS3 ura3-52 lys2-801 leu2-1 his3-Δ200*
*arp1Δ bud1Δ bud2Δ*	3674	*MATα LEU2::LEU2-GFP-TUB1 arp1Δ::KanR bud1Δ::KanR bud2Δ::HIS3 ura3-52 lys2-801 leu2-1 his3-Δ200 trp1-63*
*arp1Δ*	3681	*MAT* ***a*** * arp1Δ*::*KanR LEU*2::*LEU2-GFP-TUB1 ura3-52 lys2-801 leu2-Δ1 his3-Δ200*
*arp1Δ bud6Δ*	3697	*MAT* ***a*** * arp1Δ::KanR bud6Δ::His3MX6 LEU2::LEU2-GFP-TUB1 ura3-52 lys2-801 leu2-1 his3-Δ200*
*arp1Δ bud5Δ*	3717	*MAT* ***a*** * bud5Δ::HIS3 LEU2::LEU2-GFP-TUB1 arp1Δ::KanR ura3-52 lys2-801 leu2-1 his3-200 trp1-63*
*arp1Δ bud2Δ bud6Δ*	3960	*bud2Δ::KanMX6 bud6Δ::KanMX6 dyn1Δ::His3 GFP-TUB1::Leu2 his31 leu20 lys2-801 ura3-Δ0*
*dyn1Δ [CEN BUD2]*	5179	*MATα dyn1Δ::KanMX6 GFP-TUB1-LEU2 [pHP519 - YCp50 URA3 BUD2] his3Δ1 leu2Δ0 ura3Δ0 lys2Δ0*
*dyn1Δ [CEN bud2^F680A^]*	5180	*MATα dyn1Δ::KanMX6 GFP-TUB1-LEU2 [pHP571 - YCp50 URA3 bud2^F680A^] his3Δ1 leu2Δ0 ura3Δ0 lys2Δ0*
*dyn1Δ [CEN bud2^R682A^]*	5181	*MATα dyn1Δ::KanMX6 GFP-TUB1-LEU2 [pHP572 - YCp50 URA3 bud2^R682A^] his3Δ1 leu2Δ0 ura3Δ0 lys2Δ0*
*dyn1Δ bud2Δ [CEN BUD2]*	5182	*dyn1Δ::KanMX6 bud2Δ::KanMX6 GFP-TUB1-LEU2 [pHP519-YCp50 URA3 BUD2] his3Δ1 leu2Δ0 ura3Δ0*
*dyn1Δ bud2Δ [CEN bud2^F680A^]*	5183	*dyn1Δ::KanMX6 bud2Δ::KanMX6 GFP-TUB1-LEU2 [pHP571 - YCp50 URA3 bud2^F680A^] his3Δ1 leu2Δ0 ura3Δ0*
*dyn1Δ bud2Δ [CEN bud2^R682A^]*	5184	*dyn1Δ::KanMX6 bud2Δ::KanMX6 GFP-TUB1-LEU2 [pHP572 - YCp50 URA3 bud2^R682A^] his3Δ1 leu2Δ0 ura3Δ0*

Point mutations of *BUD2* were produced by PCR-mediate mutagenesis using a YCp50 plasmid carrying wt *BUD2*, pHP519, as template [13]. pHP571 and pHP572 carry *bud2^F680A^* and *bud2^R682A^*, respectively.

Activation and failure of the spindle position checkpoint were assayed with time-lapse video microscopy observing the behavior of the mitotic spindle in individual living cells expressing GFP-tubulin (GFP-Tub1), as described [2], [26]. In brief, living cells from asynchronous cultures were imaged, at 30°C. The observer identified cells with long, i.e. late-anaphase, spindles in the mother. These cells were followed with time-lapse fluorescence microscopy. In many cases, the spindle entered the neck and then broke down, indicating mitotic exit. In other cases, the spindle remained intact and in the mother, while in other cases the spindle broke down in the mother. The cells were followed for a period of time equal to the mean plus two standard deviations of the duration of normal mitotic exit (generally 25–35 minutes). Cells in which the spindle broke down in the mother were scored as defective for the checkpoint, while cells that remained arrested with an intact spindle in the mother were scored as normal for the checkpoint. When the spindle entered the neck, these cells were not discarded for the calculation of checkpoint integrity.

To assay bud site selection, haploid cells carrying each plasmid were grown to mid-log phase in rich liquid medium lacking uracil, and then stained with Calcofluor, as described [27]. Bud-site-selection integrity was calculated as the percentage of cells in which all bud scars were adjacent to one another, which is the normal axial pattern for haploids [7]. In the experiment shown in Figure 2C, cells with two or more bud scars were counted. In another set of experiments, we counted cells with three or more bud scars; similar results were obtained except that a higher percentage of cells exhibited the axial budding pattern with the wild-type *BUD2* plasmid.

For GAP assays, the proteins were a kind gift of Drs. Paul Polakis and Frank McCormick (Onyx Pharmaceuticals) except for GST-Bud2, which was purified from yeast in our lab. The assay conditions were as previously described, except that only one time point was measured after 20 min incubation (see Fig. 2b, Ref [10]). Briefly, Rsr1 or Ras2 was preloaded with [**γ** -^32^P]GTP, and then incubated with GST or GST-Bud2 at 23°C for 20 min, followed by a filter binding assay to measure the protein-bound radioactivity. We calculated and plotted the percent of GTP remaining, compared to the amount at time zero.

## Supporting Information

Figure S1 **Bud2 acts as a GAP for Rsr1/Bud1 but not for Ras2.** Rsr1/Bud1 or Ras2 preloaded with [γ -^32^P]GTP was incubated with GST-Bud2 or GST, and the percentage of radiolabelled GTP remaining bound to each GTPase is plotted. This plot represents an average of two experiments with similar results.
(TIF)

Movie S1 **Mitotic exit, marked by breakdown of the mitotic spindle, occurs in a** ***bud2*** **mutant cell in which a cytoplasmic microtubule is present in the mother-bud neck.**
(MOV)
