# Supplementary figures and images for: A Novel Role for the GTPase-Activating Protein Bud2 in the Spindle Position Checkpoint
Source: PLoS One. 2012 Apr 25;7(4):e36127. doi: 10.1371/journal.pone.0036127 (PMC3338500; doi:10.1371/journal.pone.0036127)

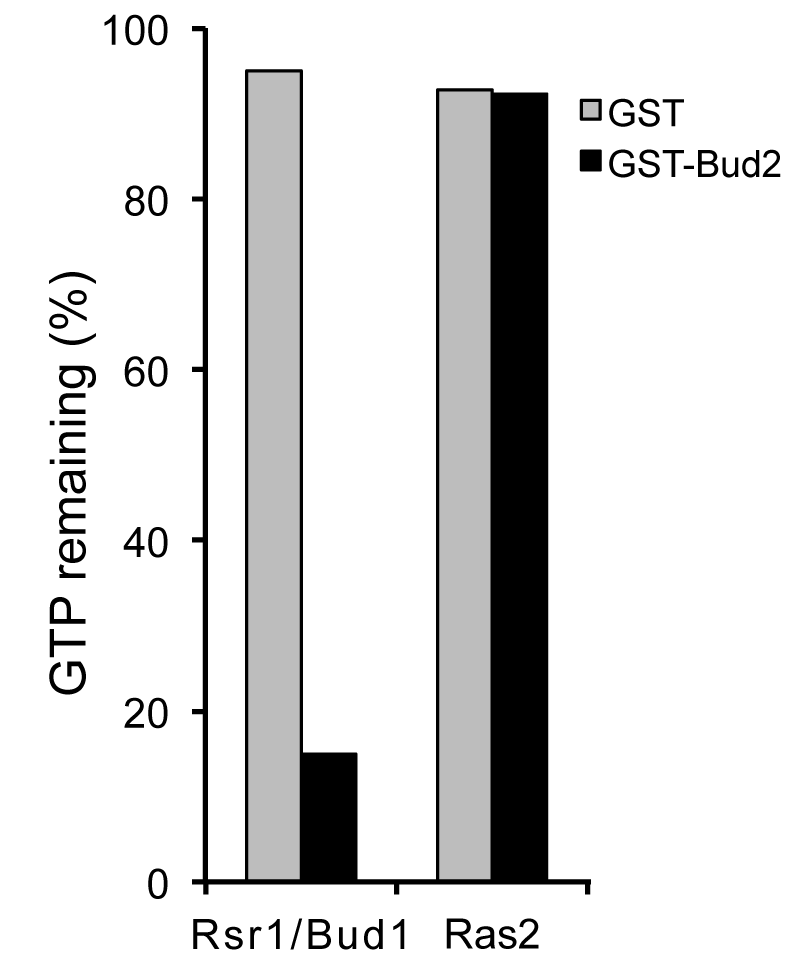

Supplement: Figure S1 — Bud2 acts as a GAP for Rsr1/Bud1 but not for Ras2. Rsr1/Bud1 or Ras2 preloaded with [γ -32P]GTP was incubated with GST-Bud2 or GST, and the percentage of radiolabelled GTP remaining bound to each GTPase is plotted. This plot represents an average of two experiments with similar results. (TIF) [file pone.0036127.s001.tif]
